# Biomarkers of Oxidative Stress in COVID-19 Patients

**DOI:** 10.3390/ijms26083869

**Published:** 2025-04-19

**Authors:** Elitsa Pavlova, Petar Atanasov, Ivaylo Ivanov, Georgi Dyankov

**Affiliations:** 1 Faculty of Physics, Sofia University “St. Kliment Ohridski”, 1164 Sofia, Bulgaria; 2 University Multiprofile Hospital for Active Treatment and Emergency Medicine “N. I. Pirogov”, 1606 Sofia, Bulgaria; gemel@mail.bg (P.A.); ivo0816@gmail.com (I.I.); 3 Institute of Optical Materials and Technology, Bulgarian Academy of Sciences, 1113 Sofia, Bulgaria; ge.dyankov@gmail.com

**Keywords:** COVID-19, inflammation, biomarkers, oxidative stress, correlation

## Abstract

We focused on evaluating oxidative stress as a major mechanism of cell damage in patients with COVID-19 infection by simultaneously assessing standard oxidative stress biomarkers in vivo—for the very first time in this specific combination—alongside typical clinical biomarkers of inflammation. Standard biomarkers were used to evaluate the oxidative stress status and antioxidant activity in the blood plasma of COVID-19 patients and healthy controls. These included TBARSs (Thiobarbituric Acid-Reactive Substances), SOD (Super Oxide Dismutase), CAT (catalase), GRA (glutathione reductase) activities, and AOC (antioxidant capacity). All clinical inflammation data confirmed a highly activated immune response in the tested COVID-19 patients: WBCs (white blood cells) were increased by nearly 100%, LYMs (lymphocytes) increased by ~30%, CRP (C-reactive protein) rose by over 2200%, and the ESR (erythrocyte sedimentation rate) increased by ~320% compared to established maximum control levels. The results confirmed that the infection involved a free-radical-mediated damage mechanism: TBARS levels increased almost 3-fold, the AOC decreased more than 4-fold, SOD was increased nearly 5-fold, CAT was increased by 1.4 times, and GRA was suppressed by 2.5 times. COVID-19 was associated with oxidative stress and suppressed antioxidant activity. All these changes contribute to the severity of the disease, complications, and mortality in COVID-19 patients.

## 1. Introduction

Severe acute respiratory syndrome-related coronavirus 2 (SARS-CoV-2) causes severe systemic symptoms [1,2,3,4]. Its pathogenesis also involves injury to cells that are not infected by the virus. This is due to the attack of ROS (Reactive Oxygen Species) that are generated from activated white blood cells, which are infiltrated into the virus-infected organs and spread in the body via the bloodstream [5,6]. When oxidative stress causes tissue damage in viral infections, the ROS pathways are a typical component of the immune response mechanisms [7,8].

The exact mechanisms by which the SARS-CoV-2 virus induces oxidative stress are not yet fully understood. However, it is known that the virus infects and harms respiratory tract cells, causing cytokines and chemokines to be released. These can activate immune cells like macrophages and neutrophils. These immune cells produce ROS as a part of their defense mechanisms against pathogens. However, excessive ROS production can cause tissue damage and oxidative stress. Furthermore, the virus can directly cause oxidative stress by interfering with the mitochondria’s function. Inflammatory pathways are triggered as a result, adding to the burden of oxidative stress. Oxidative stress, including reductive stress, can have many detrimental effects on the body, including inflammation, tissue damage, and cell death. Oxidative stress in COVID-19 can lead to the development of acute respiratory distress syndrome, a potentially fatal illness marked by lung inflammation and fluid buildup. It also plays a role in complications involving other systems, particularly the cardiovascular system, leading to conditions such as myocarditis, thrombosis, and arrhythmias.

Various diseases can complicate and burden the symptoms of COVID-19 infection. Hypertension and cardiovascular diseases are highlighted as critical comorbidities that elevate the risk of severe COVID-19 outcomes. The endothelial dysfunction that is commonly observed in hypertensive individuals may exacerbate the pro-thrombotic state that is induced by COVID-19, increasing the likelihood of thromboembolic complications [9].

Chronic kidney disease and chronic obstructive pulmonary disease are also associated with increased COVID-19 severity and mortality. Patients with chronic kidney disease often have an impaired immune response and are at higher risk of fluid overload and electrolyte imbalances, which can complicate the clinical management of COVID-19. Similarly, individuals with chronic obstructive pulmonary disease have pre-existing pulmonary damage and reduced lung function, making them more susceptible to severe respiratory complications when infected with SARS-CoV-2 [9]. 

Among the comorbidities, obesity stands out as a significant risk factor. Individuals with obesity often experience compromised respiratory function due to decreased diaphragmatic excursion and reduced lung volumes, which can exacerbate the respiratory challenges posed by COVID-19. Additionally, obesity is associated with chronic low-grade inflammation, characterized by elevated levels of pro-inflammatory cytokines such as interleukin-6 and tumor necrosis factor-α. This inflammatory medium may amplify the cytokine storm that is observed in severe COVID-19 cases, leading to acute respiratory distress syndrome and multi-organ failure. Furthermore, adipose tissue expresses the angiotensin-converting enzyme 2 receptor, which the virus utilizes for cellular entry [9].

Diabetes mellitus is another prevalent comorbidity that significantly impacts COVID-19 severity. The underlying mechanisms include chronic inflammation, impaired immune response, and potential direct pancreatic damage by SARS-CoV-2, leading to dysregulated glucose metabolism. Hyperglycemia itself can impair neutrophil function and reduce complement activity, further compromising the host’s ability to combat the viral infection [9].

The early identification of high-risk individuals through detailed medical history and appropriate diagnostic evaluations is crucial for implementing targeted therapeutic strategies.

In particular, ROS, such as hydrogen peroxide and hydroxyl radicals, superoxide anion radicals, singlet oxygen, and other free oxygen radicals that are produced in all free radical conditions and diseases are considered reagents that attack the cell membranes and give rise to lipid peroxidation [1,10]. Numerous studies have identified lipid peroxidation as a major mechanism underlying cell membrane degradation and damage.

Cellular injury or organ dysfunction caused by oxidative stress occurs when ROS accumulate in excess and damage the host defense mechanisms. These effects include the oxidation of proteins and lipids, which leads to the loss of cell membrane integrity and barrier dysfunction; cytoskeleton fragility and disrupted cell signaling; damage to the nuclear membrane and genetic material; immunological overload, which causes both acute and chronic diseases; and imbalance in the concentrations of various metal–protein complexes and the cell redox status [1,11,12,13].

The body naturally uses the free radical processes to metabolize oxygen and the harmful byproducts of metabolism, immunological response, and regeneration. The antioxidant systems typically maintain a balance between the synthesis of free radicals and ROS and their neutralization. Oxidative stress is defined as the disturbance of this equilibrium [1,14,15]. It is generally accepted that free radical damage processes are the cause of more than 60 distinct diseases [16,17,18].

Enzymes like superoxide dismutase (SOD), glutathione peroxidase (GPx), glutathione reductase (GR), and catalase (CAT) are key components of the body’s enzymatic antioxidant defense system. Non-enzymatic antioxidants include vitamin E, vitamin C, ß-carotene, uric acid, different proteins like albumin and bilirubin, and many other compounds [7,10]. In general, antioxidants are compounds that can delay, inhibit, or prevent oxidation by trapping free radicals and reducing the level of oxidative stress [17,18,19,20,21]. The body’s antioxidant capacity is continually challenged by free radical oxidation processes, particularly during the course of viral infections.

We focused on evaluating oxidative stress as a major mechanism of cell damage in patients with COVID-19 infection by simultaneously assessing standard oxidative stress biomarkers in vivo—for the very first time in this specific combination—alongside typical clinical biomarkers of inflammation.

The selected oxidative biomarkers (Thiobarbituric Acid-Reactive Substances—TBARSs), as well as the antioxidant ones (the activities of the following enzymes: superoxide dismutase, catalase, glutathione reductase, and antioxidant capacity), provided a comprehensive profile of the oxidative stress status in the patients studied. Such a complete study is very informative. All data obtained were compared to those of healthy subjects, who were not infected with SARS-CoV-2 or another infection recently and did not suffer from any chronic condition.

TBARSs are formed as a byproduct of the lipid peroxidation caused by the induced oxidative burst resulting from the infection. They are accepted as a standard biomarker. Because ROS have extremely short half-lives, their direct measurement is difficult. Instead, several products of the damage can be measured, such as TBARSs. Malondialdehyde (MDA) is one of the several low-molecular-weight end products that are formed via the decomposition of certain primary and secondary lipid peroxidation products, measurable as TBARSs [22]. That test displays the total amount of free radical peroxidation products, as well as the oxidation process trend in the infection’s target organs.

The SOD amounts in cellular and extracellular media are essential for preventing oxidative-stress-related diseases, including viral infections [23,24]. The reaction catalyzed by SOD is extremely fast, with a turnover of 2 × 10^6^ mol^−1^s^−1^, and the presence of sufficient amounts of the enzyme in cells and tissues typically keeps the concentration of superoxide radicals very low [25]. Therefore, quantifying SOD activity is crucial to accurately characterizing a biological system’s antioxidant capacity. Notably, SOD treatment has been shown to reduce the lethal or toxic effects of influenza virus infection in mouse models, although it does not inhibit the viral replication itself [26,27,28,29].

Catalase is an enzyme that is found in practically all living creatures who are exposed to oxygen. It catalyzes the conversion of hydrogen peroxide into oxygen and water [30]. It is a crucial enzyme for shielding the cell from ROS-induced oxidative damage. One catalase molecule may convert millions of hydrogen peroxide molecules into oxygen and water per second, making it one of the enzymes with the fastest turnover rates [31]. CAT is a tetramer made from four polypeptide chains, each over 500 amino acids long [32]. It contains four Fe-containing heme groups that allow the enzyme to react with H_2_O_2_.

Glutathione reductase is a flavoprotein that catalyzes the NADP.H-dependent reduction of oxidized glutathione (GSSG) to glutathione (GSH) [33]. This enzyme is a part of the GSH redox cycle. It is responsible for the levels of reduced cellular GSH. To defend against oxidative stress, a high GSH/GSSG ratio is necessary. GPx1 is predominantly present in erythrocytes, kidneys, and the liver, and GPx4 is highly expressed in renal epithelial cells and testes. The same substrate, H_2_O_2_, is used by both GPx and CAT. However, GPx is the main source of defense against oxidative stress, because it can react with lipids and other organic hydroperoxides in an efficient manner [34,35]. Therefore, oxidative stress or other circumstances that reduce GSH in the oral, nasal, and upper airway epithelium may increase the vulnerability to viral infections.

The total antioxidant capacity reflects the cumulative action of both water-soluble and lipid-soluble antioxidants. Since various antioxidants and antioxidant enzymes function synergistically in vivo, assessing the total antioxidant capacity provides a more comprehensive overview of the body’s antioxidant status than evaluating individual antioxidants or enzymes in isolation.

By concurrently evaluating common oxidative stress biomarkers in vivo—for the first time in this particular combination—alongside common clinical biomarkers of inflammation, we aimed to assess oxidative stress as a primary mechanism of cell damage in patients with COVID-19 infection.

## 2. Results

### 2.1. Clinical Data (Figure 1)

The standard clinical data obtained for the inflammation biomarkers showed that all tested COVID-19 patients exhibited markedly elevated inflammatory activity:

- The number of WBCs in the healthy controls was approximately 70% compared to the established control levels, whereas in the infected individuals, it was 30% higher.
- The number of LYMs was decreased more than two times in the infected patients in comparison to the healthy individuals.

**Figure 1:**
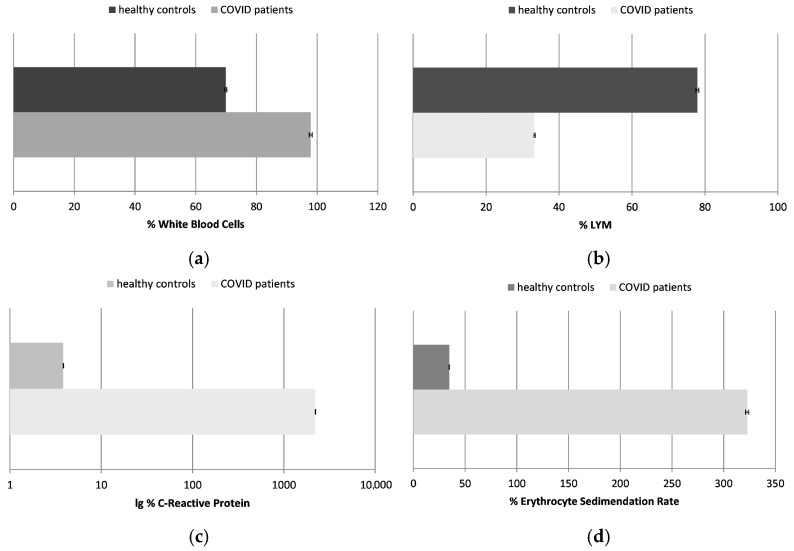
The number of white blood cells (WBCs) (**a**) and lymphocytes (LYMs) (**b**); the concentration of C-reactive protein (CRP) (**c**); and the erythrocyte sedimentation rate (ESR) (**d**), presented as a percentage in healthy control subjects and COVID-19 patients (*p* ≤ 0.05). The data are not presented in standard units due to different, but standard, methodologies and reference ranges being applied in the laboratories where the samples of the patients and healthy controls were tested; data are presented in percentages, with 100% being the maximum acceptable control value.

The white blood cells are a key component of the immune system that helps fight infections. A much higher number of WBCs is generally associated with inflammation. This was observed in the chosen patients too. In some cases, COVID-19 can cause a decrease in the number of white blood cells, especially lymphocytes, a condition called leukopenia, especially in the early stages of the disease or in severe cases. That was the case in the tested patients as well. The mechanism by which COVID-19 causes lymphopenia is not fully understood, but it may be due to a direct viral attack on the lymphocytes or to the immune response against the virus as a whole. In other cases of this infection, the opposite condition may be observed—an increase in the number of white blood cells, especially lymphocytes (leukocytosis). This increase may be due to the infection-stimulated immune response fighting the virus.

- The most informative and sensitive parameter for inflammation processes in the body is CRP. This was raised to more than 2200% in the COVID-19 patients. Its level in the healthy control was about 4% compared to the highest control levels.

The CRP levels in the blood usually rise rapidly in response to inflammation, especially in patients with severe COVID-19 infection. They are usually high in cases of respiratory failure or other complications. CRP levels can also be used to monitor the course of COVID-19 infection and the patient’s response to the chosen treatment.

- The ESR was also much higher in the tested COVID-19 patients, by more than 320%; it was calculated to be about 35% in the healthy subjects, in comparison to the established highest control levels.

The ESR measures the rate of red blood cell sedimentation. Elevated levels are generally indicative of the presence of inflammation. It has been found that the ESR is elevated in many COVID-19 patients, particularly those with severe disease. Similar to CRP, the ESR is a useful predictor of the severity of the disease in these patients.

The inflammation markers in the COVID-19 patients included in our study were significantly altered, reflecting a heightened systemic inflammatory response. However, it is important to note that inflammation in COVID-19 can vary widely depending on the severity of the infection and the individual immune responses. Our findings confirmed a strong, direct correlation between the inflammation status and oxidative stress levels in these patients, reinforcing the role of both mechanisms in the pathophysiology of the COVID-19 disease.

### 2.2. Thiobarbituric Acid-Reactive Substances (TBARSs) (Figure 2)

Our data showed that TBARSs increased by almost three times in the COVID-19 patients in comparison to the healthy controls. Elevated levels of TBARSs indicate oxidative stress, and our studies confirmed that those products accumulated in the bloodstream as a result of the infection. Thus, further exacerbated cellular damage may easily contribute to the severity of the disease.

**Figure 2:**
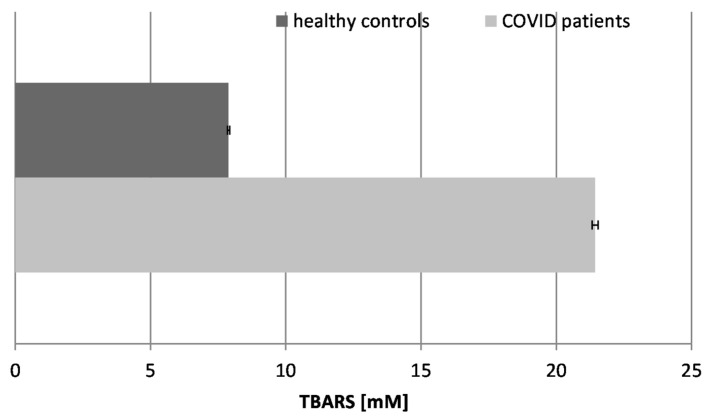
Concentration of TBARSs in the blood plasma of COVID-19 patients and healthy control subjects (*p* ≤ 0.05).

High levels of TBARSs have been found in COVID-19 patients and have been associated with disease severity and thrombotic events. These findings suggest that oxidative stress may play a role in the pathogenesis of COVID-19 and that antioxidant therapies may be beneficial in the management of COVID-19 [36]. Some results of other authors have also shown that the TBARS levels in erythrocytes are higher in COVID-19 patients than in control groups, showing the existence of an oxidative imbalance in these patients and the overproduction of free radicals [37].

### 2.3. Antioxidant Capacity (AOC) (Figure 3)

The antioxidant capacity (AOC) and activity refer to the ability of the body to neutralize harmful free radicals and ROS and to protect against oxidative stress. Some authors have reported that the total antioxidant capacity levels are considerably lower in patients compared to healthy individuals (*p*  <  0.05), including patients with mild and severe disease (*p*  <  0.05). These findings suggest that COVID-19 patients may be susceptible to a depleted total antioxidant capacity. Moreover, observing such variations in the blood samples of infected individuals could be considered a predictive marker of COVID-19 severity [38]. The antioxidant capacity reflects the overall antioxidant content and activity of the body in fighting oxidative stress of various etiologies. Our results showed that the AOC was greatly reduced in the blood plasma of COVID-19 patients by more than 4-fold, which proves the overall exhaustion of antioxidants and antioxidant enzymes in the organism. The drastic reduction in AOC indicates that the overall antioxidant capacity is overwhelmed, failing to neutralize the excess free radicals.

**Figure 3:**
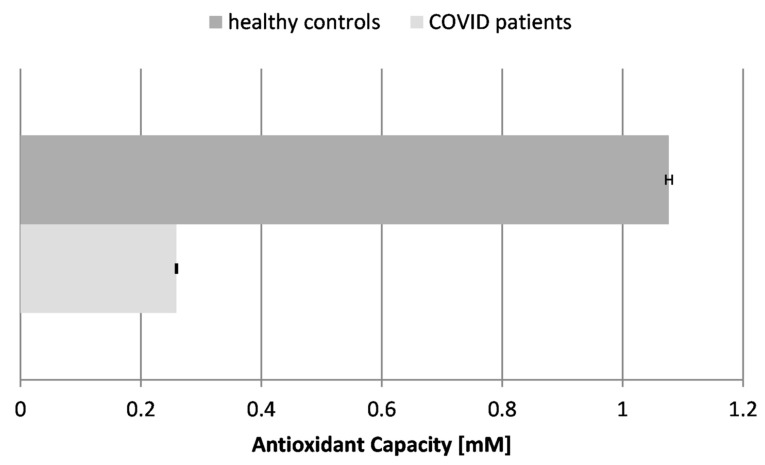
Antioxidant capacity (AOC) in the blood plasma of COVID-19 patients and healthy control subjects (*p* ≤ 0.05).

A major part of the antioxidant defense system of the organism consists of antioxidant enzymes, which are formed and kept on an ongoing basis to regulate everyday levels of oxidation in the body. We only tested the major ones—superoxide dismutase (SOD), catalase (CAT), and glutathione reductase (GR). They work together and neutralize specific ROS, maintaining the cellular redox balance.

### 2.4. Activity of Super Oxide Dismutase (SOD) (Figure 4)

Our results demonstrated that the SOD activity in COVID-19 patients was significantly elevated, being nearly 5-fold higher than in healthy individuals. This is indicative of stimulation of the enzyme in the course of the disease and its potential to fight superoxide radicals (O_2_^.−^), generated during the inflammatory response to SARS-CoV-2 infection. The significant increase in SOD activity suggests a reactive upregulation to counteract the high levels of superoxide radicals. Decreased SOD activity may be associated with serious complications and lethal outcomes in COVID-19 patients.

**Figure 4:**
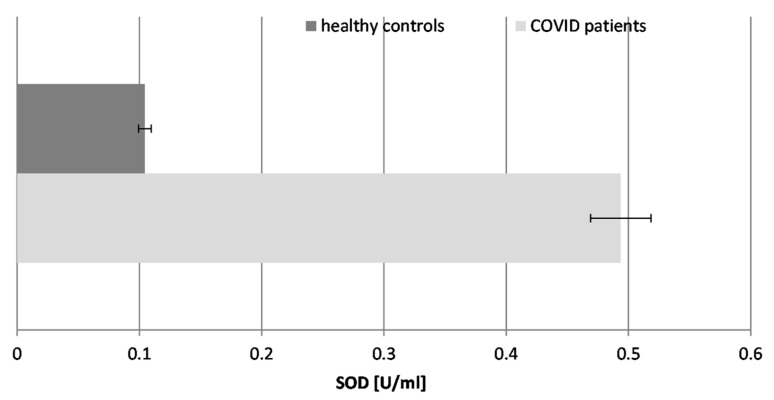
The activity of superoxide dismutase (SOD) in the blood plasma of COVID-19 patients and healthy control subjects (*p* ≤ 0.05).

SOD converts superoxide radicals (O_2_^.−^) into hydrogen peroxide (H_2_O_2_), necessitating enhanced CAT activity.

### 2.5. Catalase (CAT) Activity (Figure 5)

CAT is a key antioxidant enzyme that plays a crucial role in protecting cells from oxidative damage by catalyzing the decomposition of hydrogen peroxide (H_2_O_2_), which accumulates rapidly during disease states. Yan et al., 2020 found altered, decreased catalase activity in COVID-19 patients, suggesting that oxidative stress may contribute to the pathogenesis of the disease. Furthermore, the authors observed an inverse correlation between catalase levels and inflammatory markers, implicating oxidative stress in the cytokine storm that is commonly seen in severe COVID-19 cases [39].

**Figure 5:**
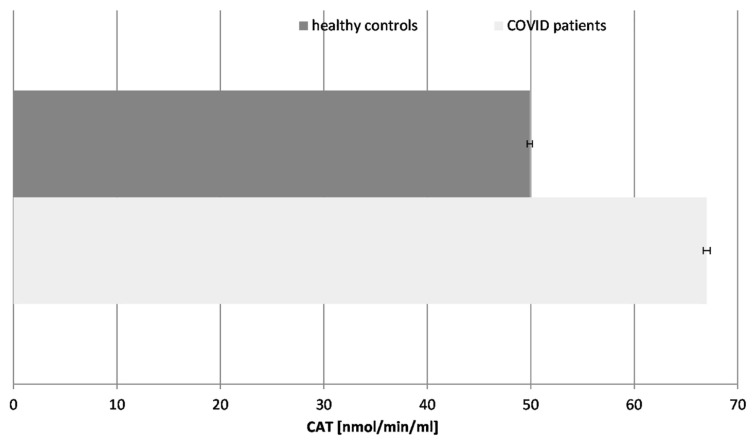
Catalase (CAT) activity in blood plasma of COVID-19 patients and healthy control subjects (*p* ≤ 0.05).

Our results revealed that the CAT activity was significantly increased (36%) in the COVID-19 patients in comparison to the healthy controls, following the pattern of the SOD enzyme. The turnover of CAT is the highest among all enzymes. The increase in CAT activity suggests an adaptive response to elevated hydrogen peroxide levels. It controls the concentration of H_2_O_2_, not only as an inflammatory but also as a messenger molecule, controlling the activity of other enzymes and genes. The activity of CAT and all antioxidant enzymes is exhausted over time, especially in COVID-19 cases with additional complications, as has been confirmed by many authors. It should be noted that all patients tested in that research were chosen during active COVID-19 infection, without any additional acute or chronic conditions or diseases.

The elevated SOD and CAT activity may provide temporary protection against oxidative damage during the acute phase of infection, limiting lipid peroxidation and cellular injury. This is supported by studies showing enhanced antioxidant enzyme activity in the early disease stages as part of systemic defense mechanisms. However, prolonged stimulation may lead to enzyme exhaustion, particularly in severe cases or patients with complications [40,41].

### 2.6. Glutathione Reductase Activity (GRA) (Figure 6)

Glutathione is a tripeptide composed of glutamate, cysteine, and glycine and appears to be a crucial antioxidant molecule in the body. It acts as a substrate for glutathione reductase and peroxidase and is also involved in detoxifying ROS and other toxins. Glutathione reductase activity refers to the rate at which this enzyme catalyzes the reduction of oxidized glutathione (GSSG) to its reduced form (GSH), using the cofactor NADPH. Glutathione reductase activity is important for maintaining the levels of reduced glutathione within cells, which can scavenge ROS and protect cells from oxidative damage. Deficiencies in glutathione reductase activity have been associated with various pathological conditions, including neurodegenerative diseases, liver dysfunction, cancer, etc. Therefore, measuring glutathione reductase activity is an important tool in assessing the oxidative stress status of cells and tissues. The suppression of GRA indicates a compromised detoxification pathway for hydrogen peroxide and lipid peroxides.

**Figure 6:**
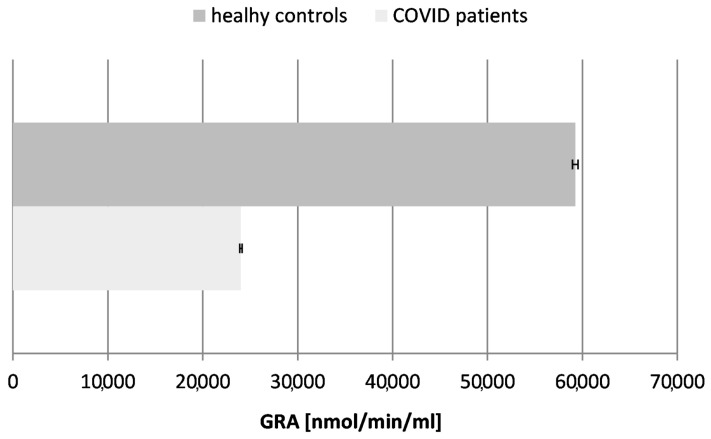
Glutathione reductase activity (GRA) in blood plasma of COVID-19 patients and healthy control subjects (*p* ≤ 0.05).

Our results confirmed a ~2.5-fold decrease in the activity of glutathione reductase in the tested COVID-19 patients in comparison to the controls (healthy subjects), proving the exhaustion of glutathione as a major antioxidant molecule in the organism during and after the acute phase of the infection.

The contrasting trends, the upregulation of SOD and CAT and downregulation of GRA, reflect a dynamic interplay between compensatory mechanisms and exhaustion phases:

- Early adaptive response: During the initial infection stages, increased SOD and CAT activities mitigate ROS accumulation, protecting cellular components from oxidative damage
- Progressive exhaustion: As the infection persists, antioxidant systems like GRA become depleted due to sustained ROS production and inflammation. This exhaustion phase reduces the cellular resilience against oxidative stress, potentially contributing to complications such as organ failure or severe systemic inflammation.

Monitoring SOD, CAT, and GRA activities could serve as biomarkers for disease progression or therapeutic efficacy. Elevated SOD and CAT may indicate early compensatory responses, while reduced GRA could signal impending oxidative-stress-related complications [40,41].

### 2.7. Correlation of Clinical and Experimental Biomarkers Between Healthy and Infected Subjects

The analysis of the clinical parameters (number of white blood cells, % lymphocytes, C-reactive protein, and erythrocyte sedimentation rate) as standard clinical inflammatory markers and the measured basic parameters of oxidative status in the organism (TBARSs, AOC, SOD, CAT, GRA) showed complete correlation between the data of the tested healthy subjects and the COVID-19 patients (r = +0.995), (Figure S1). The used function computes the Pearson correlation coefficient, which measures the strength and direction of the linear relationship between two variables. This is another confirmation that oxidative stress and the free radical and oxidative mechanism, on the one hand, and the cytokine storm, on the other, appear to be a damaging pathway in patients with “pure” COVID-19 infection.

A major strength of our study lies in the simultaneous assessment of multiple standardized biomarkers with strong clinical relevance, allowing for a comprehensive overview of both oxidative and inflammatory processes. However, a key limitation is that although our findings establish a robust correlation, they do not provide direct evidence of causation. The exact mechanisms by which oxidative stress may drive disease severity in COVID-19 remain to be elucidated through targeted experimental studies.

A cytokine storm, a severe immune response marked by excessive cytokine production, can arise not only from viral evasion strategies but also from genetic abnormalities in the host. Cytokines contribute to endothelial permeability by damaging junctional proteins like VE-cadherin or suppressing their synthesis. SARS-CoV-2 has been shown to activate Toll-like receptor (TLR) 9 and NF-κB, which increases the expression of inflammatory genes while reducing nitric oxide, a key vasodilator and antithrombotic agent. Additionally, SARS-CoV-2 can hyperactivate neutrophils via the NLR family pyrin domain containing 3 (NLRP3), causing leukocytes to release ROS, extracellular traps (NETs), and proteolytic enzymes that perpetuate inflammation [42].

SARS-CoV-2 also drives chronic inflammation through mitochondrial dysfunction and the activation of the cyclic GMP-AMP synthase (cGAS)-stimulator of the interferon genes (STING) pathway. Elevated circulating inflammatory agents, endothelial dysfunction, and micro-thrombosis increase the risk of cardiovascular complications, including the progression of atherosclerotic plaques, arterial or venous thrombosis, and heightened arterial stiffness [42].

SARS-CoV-2 enters the host cells primarily through the ACE2 receptor. The spike (S) protein plays a crucial role in this process. The host proteases, such as TMPRSS2 and cathepsins B and L, facilitate the viral entry by activating the S protein.

Based on these factors, the possible therapeutic strategies for COVID-19 may include the following:

- Protease inhibitors that target host proteases like TMPRSS2 and cathepsins, blocking the virus’s entry;
- Antiviral agents—drugs inhibiting the viral RNA replication by various mechanisms;
- Immunomodulators, such as JAK inhibitors, reducing the inflammatory response;
- Plasma therapy—the application of antibodies from recovered patients to stimulate the immune response;
- Monoclonal antibodies—engineered antibodies targeting viral proteins, e.g., the spike protein;
- Vaccines, which are more effective against SARS-CoV-2 and its variants;
- Antioxidants—a supportive therapy against the inflammation and oxidative stress in the organism [43].

The free radical mechanism is a basic damaging pathway. It has been proven to have great clinical significance. Cell damage and oxidative stress contribute to the disease severity, complications, and mortality in COVID-19 patients [44]. Therefore, antioxidant agents targeting oxidative stress are suitable for reducing its severity [45]. The combinations of antioxidants and antiviral drugs could synergistically reduce the complications and lethal effects of the infection. Further research is needed to explore targeted antioxidant therapies as potential adjunct treatments for COVID-19 to mitigate oxidative damage and improve the clinical outcomes.

The elucidation of the mechanisms of prophylaxis, damage, and therapy in this type of viral infection is of great importance and social significance. Despite the number of articles available on the topic, the information reported by other authors on the damage mechanisms is still insufficient or missing for many specific reactions, products, and activities. The simultaneous evaluation of the chosen inflammatory and oxidative major biomarkers investigated in our research clarified, to some extent, the role of oxidative stress in COVID-19.

## 3. Materials and Methods

### 3.1. Human Subjects

Blood and blood plasma were obtained and tested on the same day from individuals who were eligible for participation: 27 hospitalized male adults (n = 27), aged 23–62, with a confirmed COVID-19 diagnosis and active, severe infection, and 25 healthy male and 5 healthy female individuals (same age range, n = 30), assigned to the tested healthy group. All COVID-19 patients had recovered for shorter or longer periods in the hospital. No antioxidant supplements or therapy were applied to any of the tested people. The exclusion criteria included all other acute or chronic diseases or conditions and accompanying medications. All these restrictions significantly reduced the number of chosen patients and control individuals.

The gender imbalance (100% male patients vs. 83% male controls) between the two tested groups may affect the antioxidant responses—estrogen enhances glutathione synthesis, meaning a better antioxidant defense and more stable control levels, while testosterone may suppress immune responses, meaning lower oxidative stress levels. Nevertheless, the differences registered between both groups are significant and credible.

The tested blood plasma was stored frozen at −30 °C. The procedures were performed in compliance with the relevant laws and institutional guidelines and according to the ethical standards of the Declaration of Helsinki and preliminarily approved by the Ethics Committees of the involved institutions (ДК1/10 29 March 2021). Informed consent was obtained from every individual taking part in the research.

### 3.2. Clinical Data

The standard clinical data used for the number of white blood cells (WBCs), C-reactive protein (CRP) content, erythrocyte sedimentation rate (ESR), and number/percentage of lymphocytes (LYMs) were obtained from the University Multiprofile Hospital for Active Treatment and Emergency Medicine “N. I. Pirogov”, (COVID-19 patients), the National Center for Transfusion Hematology and the Diagen Lab (healthy controls), Sofia, Bulgaria (Table 1).

### 3.3. Reagents

TBARSs, SOD, CAT, GR activities, and AOC were measured using kits from Cayman Chemicals, Ann Arbor, MI, USA, following the manufacturer’s protocols. Detailed information on each method, including reagents and its protocol, can be found at https://www.caymanchem.com/products/categories and the following links that were first visited on 18 May 2020:

TBARS Assay Kit, Cat. # 10009055
https://cdn.caymanchem.com/cdn/seawolf/insert/10009055.pdf
SuperOxide Dismutase Assay Kit, Cat. # 706002
https://cdn.caymanchem.com/cdn/seawolf/insert/706002.pdf
Catalase Assay Kit Cat. # 707002
https://cdn.caymanchem.com/cdn/seawolf/insert/707002.pdf
Glutathione Reductase Assay Kit, Cat. # 703202,
https://cdn.caymanchem.com/cdn/seawolf/insert/703202.pdf
Antioxidant Assay Kit, Cat. # 709001
https://cdn.caymanchem.com/cdn/seawolf/insert/709001.pdf

Spectrophotometrically, all samples were measured by SPECTROstar^®^ Nano, BMG LABTECH, Ortenberg, Germany.

### 3.4. Statistical Analysis

The limited statistical power in this study poses risks of Type II errors, inflated effect sizes, and reduced generalizability, while introducing confounding factors like a gender imbalance. We employed strategies to improve the power and reduce bias during the measurement, data analysis, and interpretation stages. All results were obtained as triple reproducible measurements. All random and systematic errors were excluded. Statistical analysis (Student’s *t*-test) was performed using Origin 8.5 (OriginLab, Northampton, MA, USA) and Microsoft Office Excel 2010 (Microsoft Corp., Redmond, WA, USA) software. Data were accepted as statistically significant at *p* ≤ 0.05.

## 4. Conclusions

All clinical biomarkers of inflammation confirmed the highly activated status of the immune system in the tested COVID-19 patients: WBCs were increased by almost 100%, LYMs were increased by ~30%, CRP was raised by more than 2200%, and ESR was increased by ~320% in comparison to the established maximum control levels.

The biomarkers of oxidative stress confirmed that the infection possesses a free radical damage mechanism: TBARSs were increased almost 3-fold, AOC was reduced more than 4-fold, SOD was increased almost 5-fold, CAT was increased 1.4-fold, and GRA was suppressed 2.5-fold in the patients with “pure” COVID-19 infection. None of the tested COVID-19 patients suffered from any other acute or chronic conditions.

In conclusion, COVID-19 is associated with oxidative stress and suppressed antioxidant activity. COVID-19 infection can be described as a free radical disease.

## Figures and Tables

**Table 1:** Reference ranges of the tested clinical inflammation biomarkers.

Reference Ranges	UMHATEM “N. I. Pirogov” Lab	Diagen Lab
WBCs [×10^9^]	4.1–11	3.5–10.5
LYMs	0.6–4.1 [×10^9^]	20–40%
CRP	0.0–0.5 [mg/dL]	0.00–10.00 [mg/L]
ESR [mm/h]	0–15	2–20

## Data Availability

All research data are available upon request.

## Supplementary Materials

The following supporting information can be downloaded at https://www.mdpi.com/article/10.3390/ijms26083869/s1.

## Abbreviations

The following abbreviations are used in this manuscript:

COVID-19	Coronavirus Disease of 2019
TBARSs	Thiobarbituric Acid-Reactive Substances
SOD	Super Oxide Dismutase
CAT	Catalase
GRA	Glutathione Reductase Activity
AOC	Antioxidant Capacity
WBCs	White Blood Cells
LYMs	Lymphocytes
CRP	C-Reactive Protein
ESR	Erythrocyte Sedimentation Rate
SARS-CoV-2	Severe Acute Respiratory Syndrome-Related Coronavirus 2
ROS	Reactive Oxygen Species
GPx	Glutathione-Peroxidase
GR	Glutathione-Reductase
MDA	Malondialdehyde
GSSG	Oxidized Glutathione
GSH	Glutathione
