# Supplementary material for: Biomarkers of Oxidative Stress in COVID-19 Patients
Source: Int J Mol Sci. 2025 Apr 19;26(8):3869. doi: 10.3390/ijms26083869 (PMC12027644; doi:10.3390/ijms26083869)
Supplement: Supplementary file 1 [file ijms-26-03869-s001.zip › ijms-3586286-supplementary.pdf]

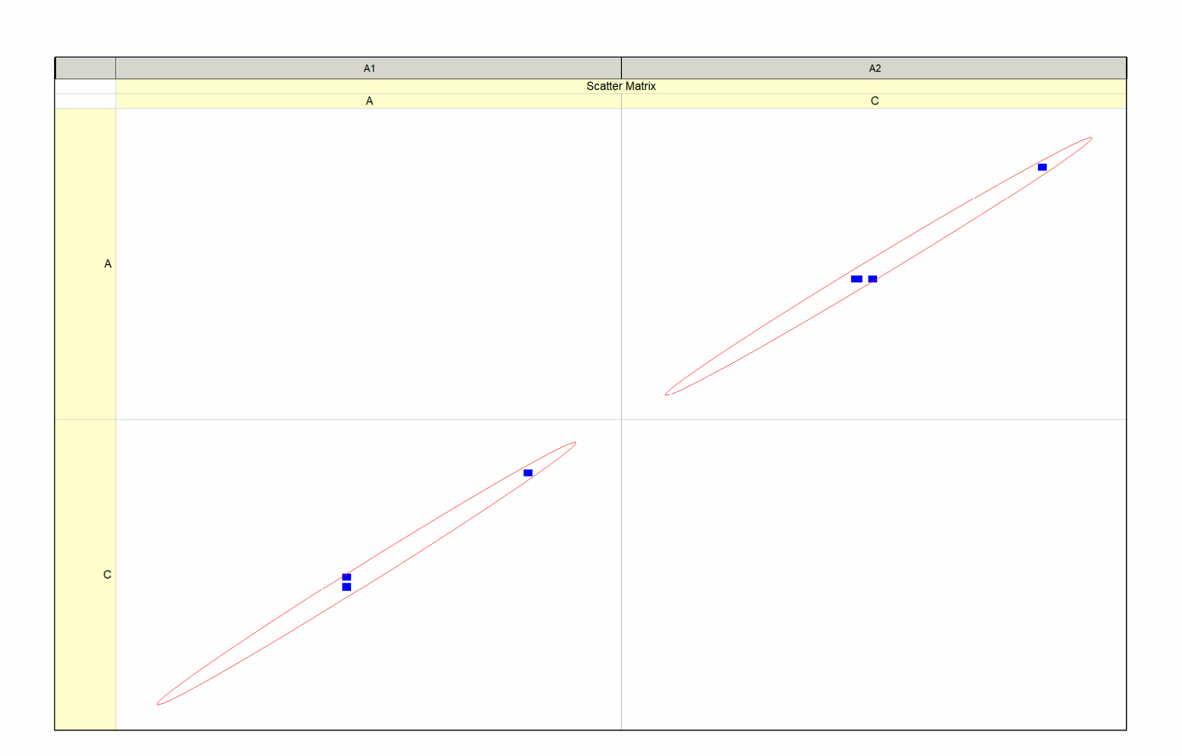

Figure S1: A scatter plot between the data sets (9 parameters per each set) – clinical and oxidative stress biomarkers, set 1 - control healthy individuals, set 2 – patients
